# Supplementary material for: DMRforPairs: identifying Differentially Methylated Regions between unique samples using array based methylation profiles
Source: BMC Bioinformatics. 2014 May 15;15:141. doi: 10.1186/1471-2105-15-141 (PMC4046028; doi:10.1186/1471-2105-15-141)
Supplement: Additional file 2 — DMRforPairs output for the comparison of A431-MCF7 and NA17018-NA17105. Please start from the HTML files in each folder. Available via the BMC Bioinformatics website. [file 1471-2105-15-141-S2.zip › 1394847754114233_MOESM2_ESM/A431_MCF7/figures/24181.pdf]

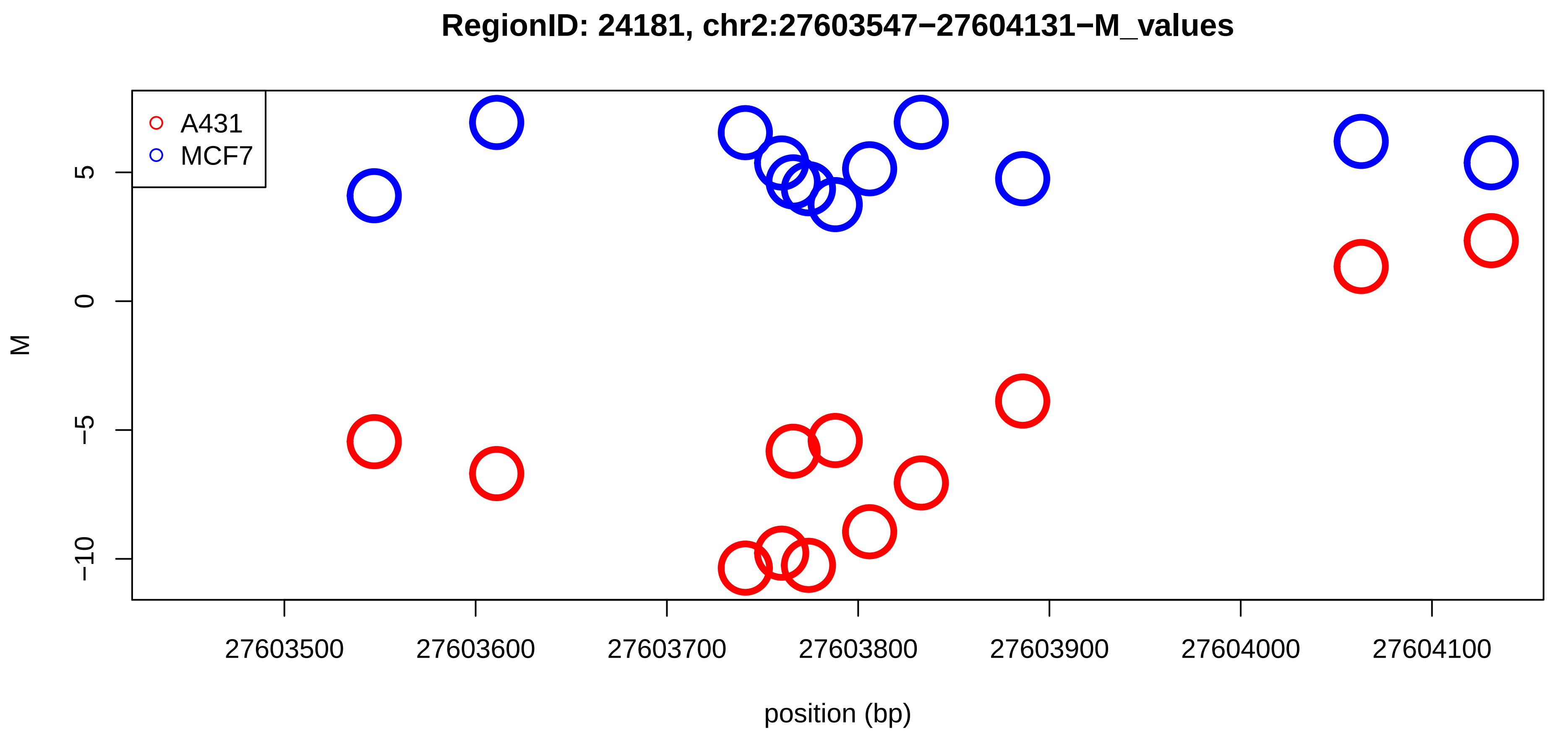
RegionID: 24181, chr2:27603547−27604131−M_values
A431
MCF7
M
5
0
−5
−10
27603500
27603600
27603700
27603800
27603900
27604000
27604100
position (bp)

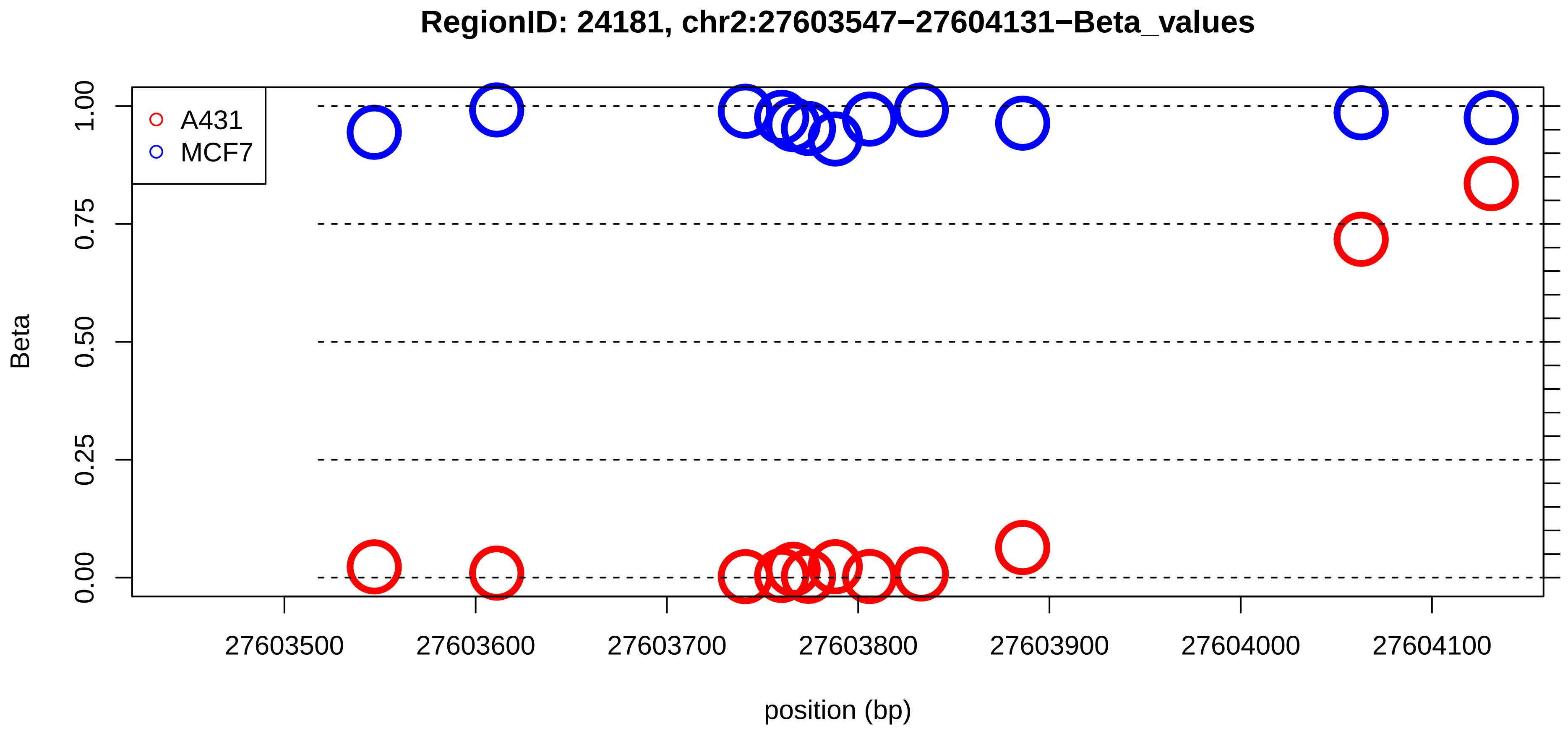
RegionID: 24181, chr2:27603547−27604131−Beta_values
A431
MCF7
Beta
1.00
0.75
0.50
0.25
0.00
27603500
27603600
27603700
27603800
27603900
27604000
27604100
position (bp)

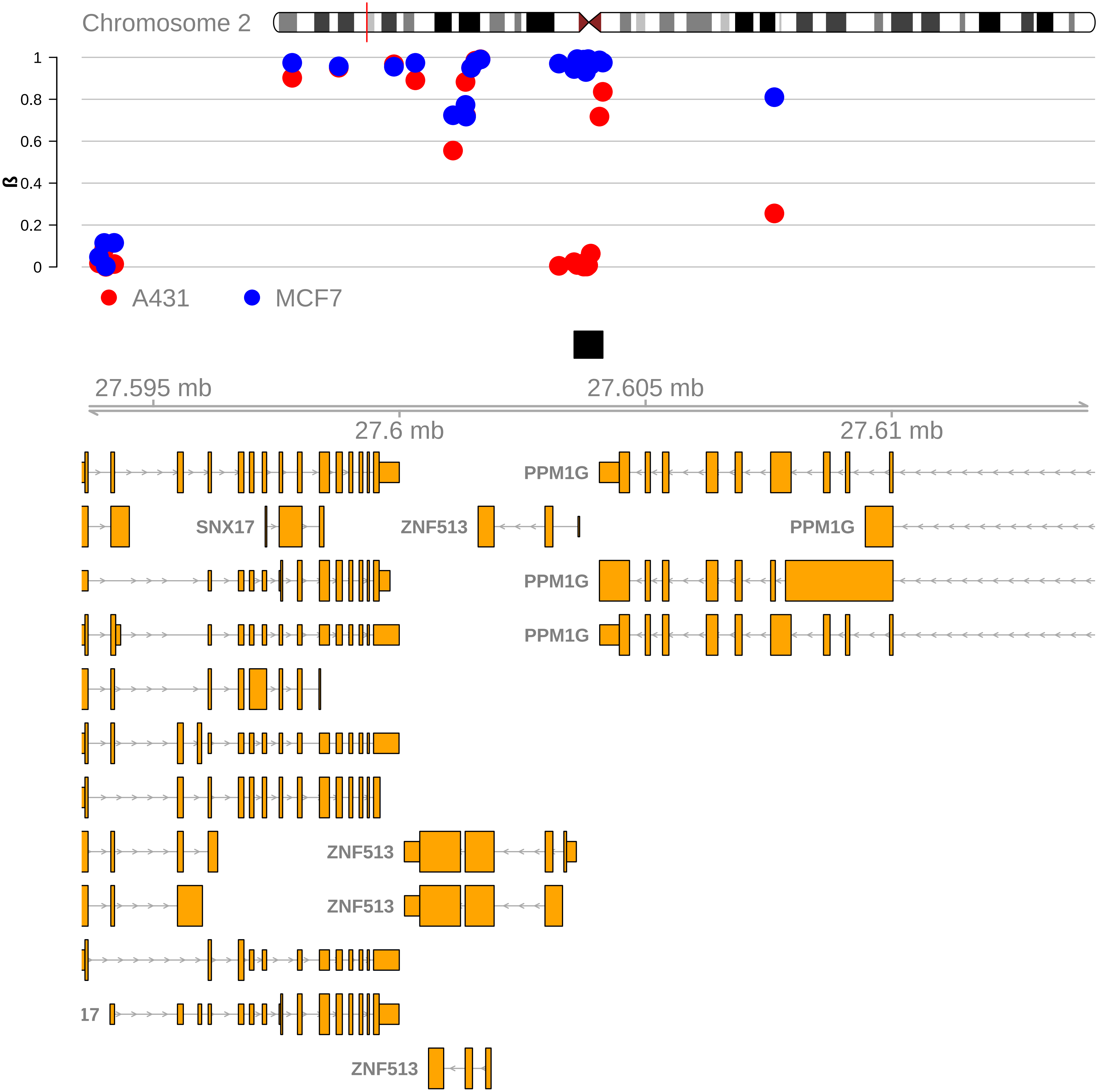

Chromosome 2
1
0.8
0.6
ß 0.4
0.2
0
A431
MCF7
27.595 mb
27.605 mb
27.6 mb
27.61 mb
PPM1G
SNX17
ZNF513
PPM1G
PPM1G
PPM1G
ZNF513
ZNF513
17
ZNF513
